# Bone wax in the treatment of partial epiphysiodesis of distal femoral growth plate: Case report at 10-year follow-up

**DOI:** 10.3389/fsurg.2022.968214

**Published:** 2022-10-18

**Authors:** Luca Basiglini, Angelo Gabriele Aulisa, Diletta Bandinelli, Renato Maria Toniolo, Francesco Falciglia

**Affiliations:** ^1^ Surgical Department, Bambino Gesù Children's Hospital, IRCCS, Rome, Italy; ^2^ University of Cassino and Southern Lazio, Cassino, Italy

**Keywords:** epiphysiodesis, growth plate, bone wax, long follow-up, case reports, physeal injury, polytrauma, growth arrest

## Abstract

The growth plate is the weakest structure in the skeleton of a child and a frequent site of injury or fracture; physeal injuries represent 15%–30% of all fractures in children. Of all growth plate fractures, the incidence of growth arrest and disorders is around 15%. Here, we discuss a female patient who, at the age of 5 years, was treated for a polytrauma that involved a complex lesion of the growth plates of the knee. Four days after trauma, she underwent closed reduction surgery and internal fixation with cannulated screws for femoral and tibial fractures of the growth plate. A 20° valgus deviation of the left knee was found at 3-month postoperative clinical check-up likely as a result of a growth disorder of the femur. She was diagnosed with valgus knee secondary to epiphysiodesis of the lateral portion of the femoral physis and she was readmitted to the hospital. In the operating theater, an open femoral de-epiphysiodesis was performed with a burr; the drilled hole was then filled with bone wax. At 20-month post-trauma follow-up, the left knee was still valgus about 20° relative to the other side. During follow-up, a slow but progressive improvement in the axis of the lower limbs was noted. Clinical and radiographic control 10 years after the trauma showed a complete recovery of the axis of the lower limbs. In the initial stages, the presence of bone wax in the area of de-epiphysiodesis allowed for stabilization of the deformity on the 20° of preoperative valgus. The interpretation of the growth cartilage activity occurred in an asymmetrical way such as to realign the femoral load axis, it can be based on the different mechanical stimulus on the two knee areas due to the preexisting deformity. There is no unanimous evidence in the literature in terms of management of growth disorders resulting from this type of injury. Bone wax resulted in effectively filling the hole of de-epiphysiodesis in the distal femoral growth plate and allowed us to obtain the response of the growth plate and to improve the recovery time in young children.

## Introduction

The growth plate is a cartilage region located at the end of the long bones of children and adolescents, which serves as the primary center for longitudinal growth and characterizes the immature skeleton; physeal lesion may result from musculoskeletal injuries including fracture, infection, malignant tumor, or iatrogenic damage (1). The growth plate is the weakest structure in the skeleton of a child and a frequent site of an injury or fracture; physeal injuries represent 15%–30% of all fractures in children (1, 2). Several factors must be considered when evaluating a child with a physis fracture such as the patient's age, location of the physis, type of fracture, and growth potential of the involved physis (2, 3). Of all growth plate fractures, the incidence of growth arrest and disorders is around 15% (4). In case of injury to a large undulating physis, as in the fracture of the distal growth plate of the femur, the plane of the fracture would cross different areas of the physis (2). Similarly, intra-articular physis fractures, such as Salter–Harris (SH) types III and IV, would traverse several or all areas of the physis. Such injuries have a worse prognosis possibility of bone formation through the physis (1). When the formation of bone bars occurs in patients with potential growth disorder, the current gold standard therapy is the resection of the bone bar with subsequent interposition of material such as free fat graft, polymethylmethacrylate (PMMA), silastic, cartilage, bone wax, and dura (2).

Here, we discuss about a 5-year-old patient treated for a polytrauma that involved a complex lesion of the growth plates of the knee and we review the treatment for subsequent deformities.

## Case report

**Figure 1:**
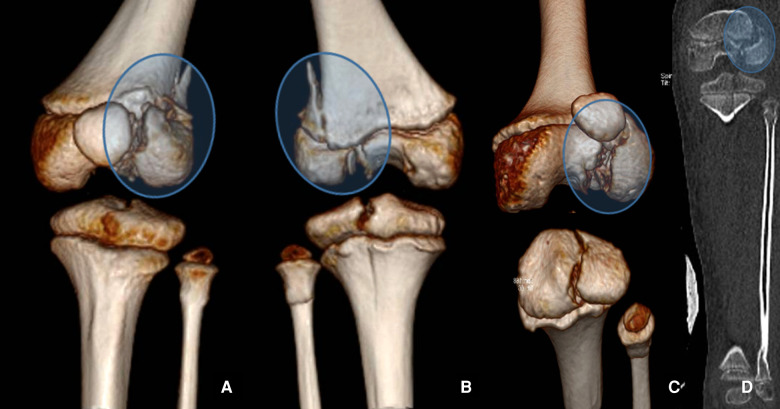
3D CT reconstruction of physis injuries of the left knee in anterior view (**A**), in posterior view (**B**), inlet view (**C**), and 2D CT coronal view (**D**).

**Figure 2:**
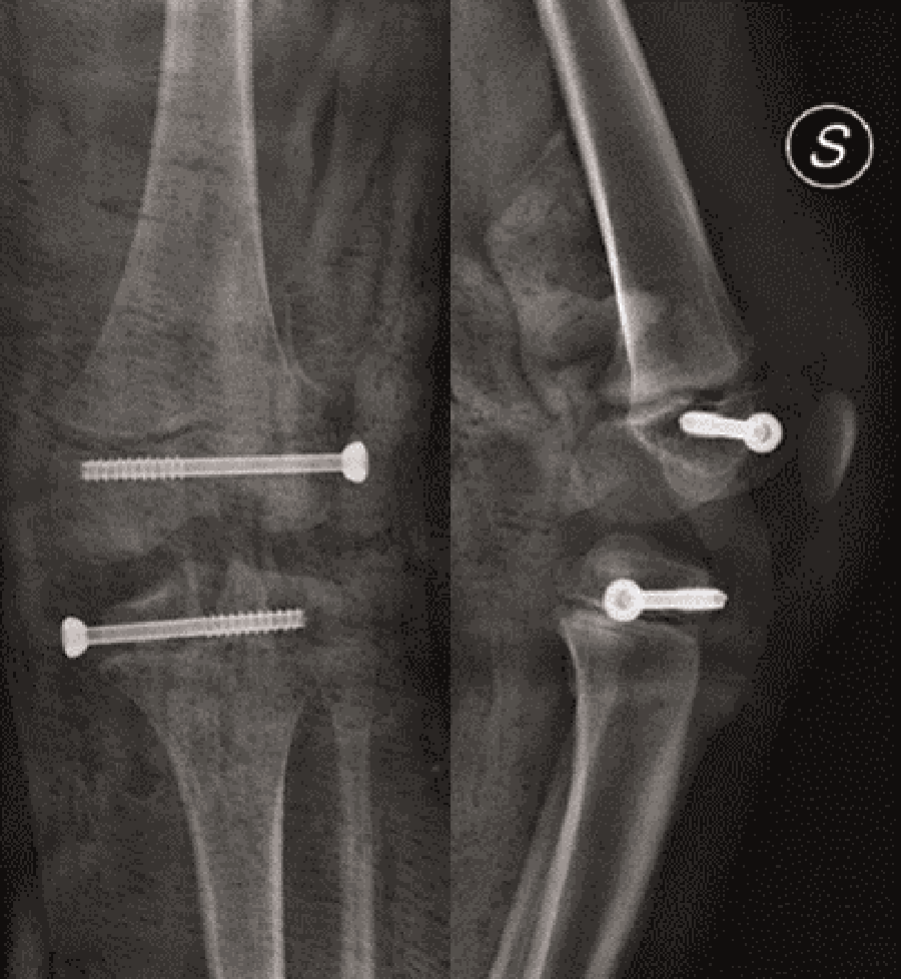
Postoperative x-ray control after reduction and synthesis of left knee growth plate fractures.

**Figure 3:**
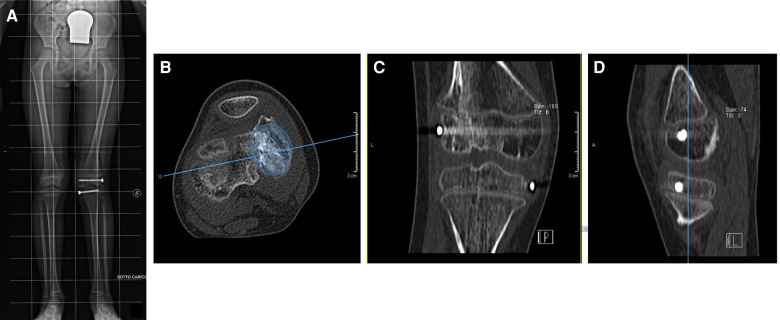
Four months postoperative x-ray showing a valgus (20°) of the left lower limb and the absence of dysmetria (**A**). CT scans confirm the presence of femoral bone bridge in three different projections (**B–D**). (**B**) shows that the bony bar is 30% of the entire area of the growth plate.

**Figure 4:**
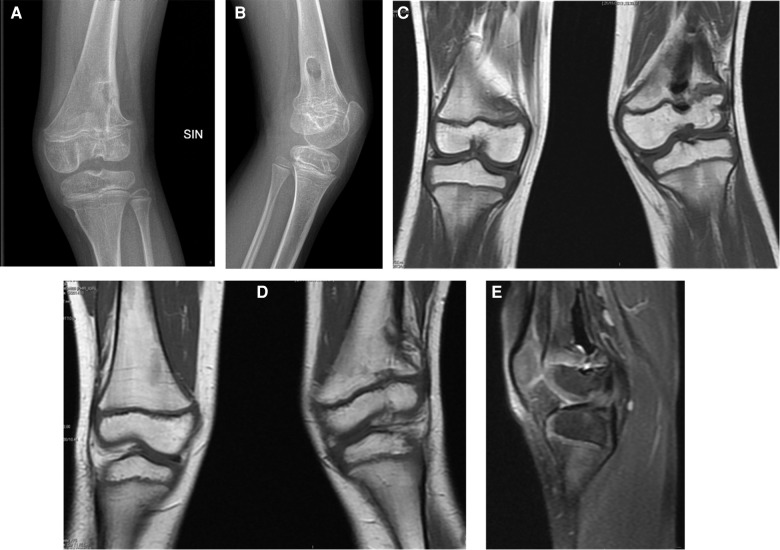
X-ray (**A,B**) and MRI (**C–E**) highlighted the results of the recent de-epiphysiodesis operation with placement of bone wax and minimal residual bone bridge.

**Figure 5:**
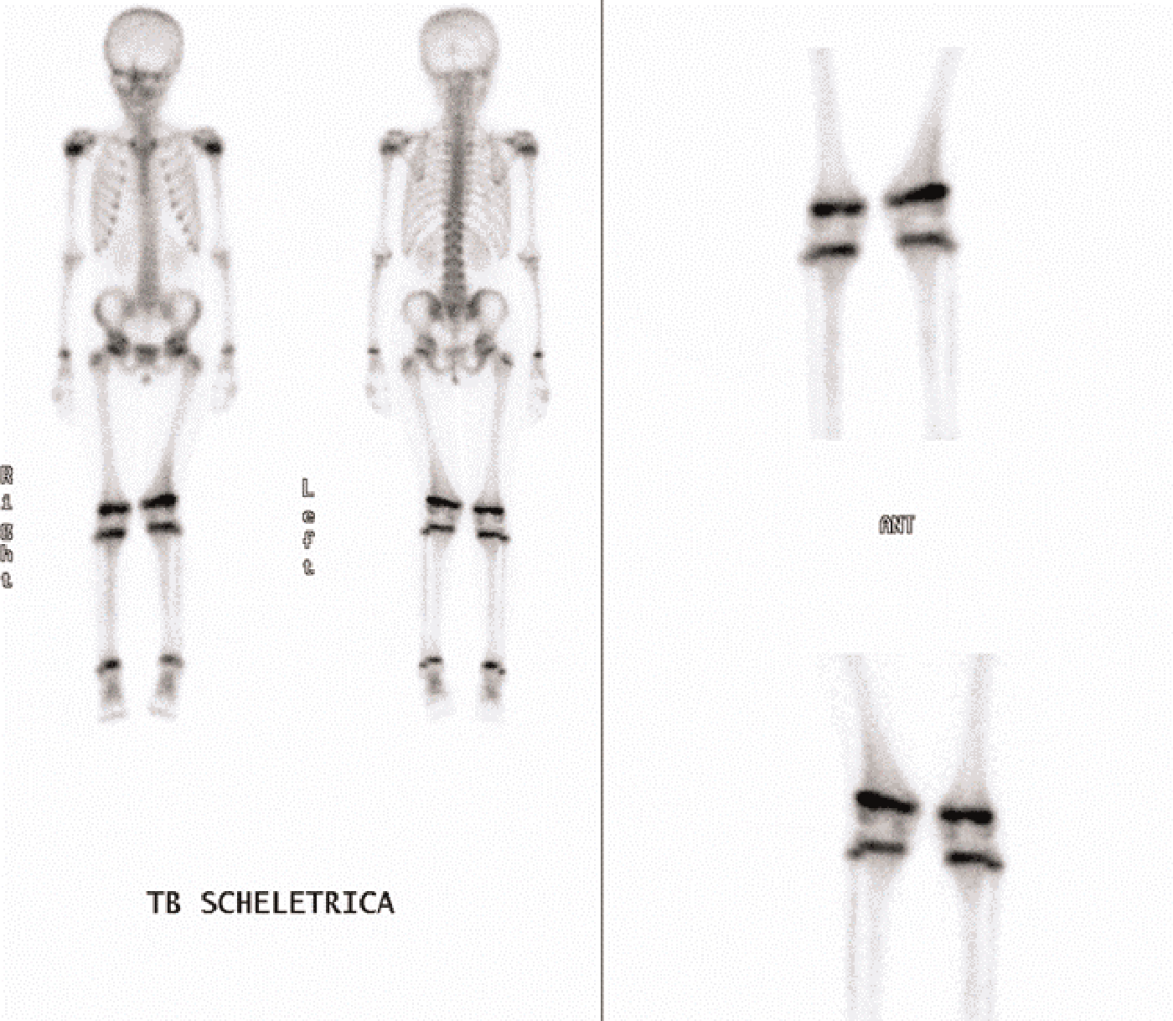
Bone scintigraphy highlighted an hypercaptation of the lateral portion of the left femoral physis.

**Figure 6:**
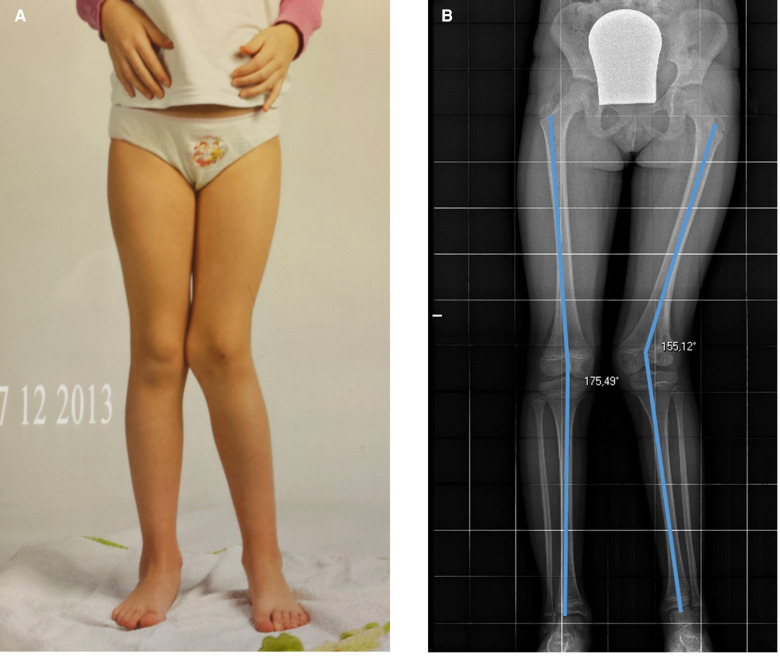
Twenty-month post-trauma follow-up showing 20° valgus deviation of the left knee with respect to the contralateral side. (**A**) showing clinical results, (**B**) showing radiological results.

**Figure 7:**
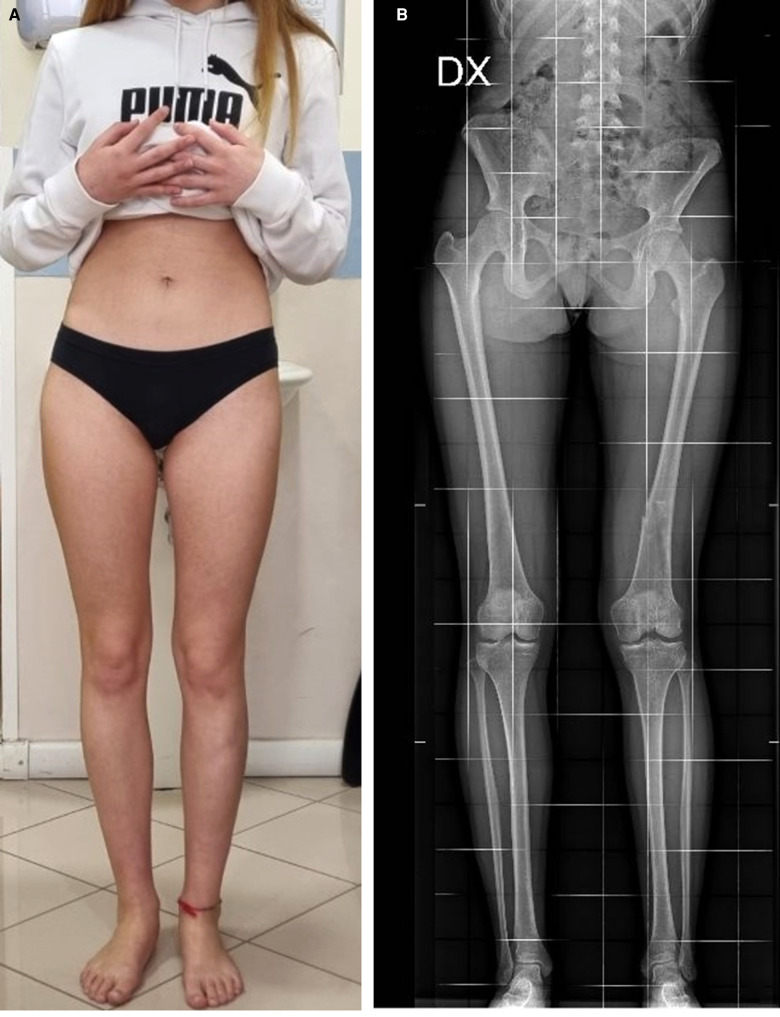
Ten-year post-trauma follow-up showing complete clinical (**A**) and radiological (**B**) recovery.

In this paper, we discuss about a 5-year-old patient overwhelmed by masonry elements following a collapse of the chimney at the patient's home in May 2012. The child was transferred from another university polyclinic to our emergency room with a diagnosis of crushing polytrauma. She had a fracture of the right ischiopubic branch, a lesion of the distal femoral and proximal tibia physis of the left knee, both type IV according to the Salter–Harris classification; a bifocal fracture of the left fibula; and diffuse torn wounds (Figure 1). The patient came in spontaneous breathing, hypotension, and tachycardia; a plasma expander was, therefore, infused and the child was then hospitalized in the Intensive Care Unit. Once stabilized, she was transferred to the Traumatology Department and underwent closed reduction surgery and internal fixation with cannulated screws for femoral and tibial fractures of the growth plate (Figure 2). After 10 days of hospitalization and treatment, the patient was discharged in good clinical condition. A weightless cast on the left lower limb was prescribed for 40 days; following a clinical-radiographic examination, the cast was then removed and kinesitherapy aimed at the gradual and complete recovery of knee movement was started. A valgus deviation of the left knee was found at 3-month postoperative clinical follow-up likely as a result of a growth disorder of the femur. Therefore, an x-ray examination of the lower limbs’ weight-bearing was performed at 4 months after operation, which showed a valgus (20°) of the left lower limb, and she presented 5 mm of limb length discrepancy. For this reason and with the diagnostic suspicion of femoral bony bar formation or growth arrest, the patient underwent a CT scan that confirmed the presence of femoral bone bridge (Figure 3). She was diagnosed with valgus knee due to partial epiphysiodesis of the femur and she was readmitted to the hospital. In the operating theater, both screws were removed percutaneously. Through a lateral surgical approach to the distal third of the left thigh, a femoral cortical bone dowel was placed near the bone bridge; then an open femoral de-epiphysiodesis with a burr from proximal to distal carefully avoiding damage to the joint surface from the inside was performed; the drilled hole was then filled with bone wax. About a month after the second surgery, the patient underwent an MRI scan that highlighted the results of the recent de-epiphysiodesis operation with placement of bone wax and minimal residual bone bridge of limited entity (2 mm × 4 mm) compared to the previous CT control (Figure 4). The patient underwent further II level investigation 18 months after trauma such as a bone scintigraphy that noticed an area of growth plate hypercaptation around the surgically performed bone hole sign of metabolic overactivity of the growth cartilage around the bone wax area (Figure 5). The young patient was then followed up over time with clinical/instrumental investigations approximately every 6 months. At 20-month post-trauma follow-up, the left knee was valgus about 20° relative to the other side with an acquired length discrepancy of 14 mm and compensatory pelvic obliquity (Figure 6). From that clinical follow-up, a slow but progressive improvement in the axis of the lower limbs was noted; the patient was then monitored over the years with clinical and radiographic follow-ups, which showed constant improvement up to complete recovery. Clinical and radiographic control 10 years after the trauma showed a complete recovery of the axis of the lower limbs associated with the absence of limitations on the left knee; in addition, the x-rays confirmed the known lowering of the left hemipelvis of about 20 mm and the known morpho-structural alteration localized at the level of the distal third shaft of the left femur as a result of the bone wax placement surgery (Figure 7).

## Discussion

Physeal injuries represent 15%–30% of all fractures in children. Fortunately, although growth plate injuries are common, growth disturbance related to physis lesions is rare, accounting for around 1%–15% of all physis injuries. There are factors that contribute to the increased likelihood of growth disturbances, such as comminuted fractures, high-energy injuries, and physeal injuries that cross the germinal layers (Salter–Harris type III and IV injuries) (5). Such injuries have a worse prognosis possibility of bone formation through the physis producing consequences like growth arrest, angular rotational deformities, and subsequent altered joint mechanics (1). As reported by Garrett et al. (6), also in this case, there was a correlation between the high-energy traumatic mechanism and the formation of the bone bridge. The type of fracture, Salter–Harris type IV, was also mostly associated with complicated risk of growth disturbance (7).

Distal femur fractures often involve the growth plate, which contributes up to 70% of the length of the femur. These kinds of fractures are known to be associated with a high incidence of complications (8). Distal femur growth disorder is the most common complication (7); growth arrest is a frequent occurrence of fractures in this region, resulting in axial and length-related deformities. Long-term surveillance is recommended to identify evolutionary deformity and provide an opportunity of early intervention (9). Adams et al. found that physeal arrest is the most common consequence following this type of injury, and when it is associated with growth disturbances such as axial and length-related deformity, it should require surgical intervention in up to 60% of cases (8).

Although it has been reported that a 50% bone bridge of the physis area can be successfully removed, 30% appears to be a threshold based on recent reports in the literature (2). Similarly, the general indications for performing hemiepiphysiodesis and osteotomy are >5° and >10° of axial deformity, respectively. This can vary according to the type of physiology, the residual growth, and the decision shared with the patient (2). Surgical resection of the bone bridge is important because the bar should be completely removed ensuring the least possible trauma to the surrounding tissues; at the same time, the bone bridge must be clearly visible and identifiable to obtain an acceptable resection (10). The techniques for removing the bar depend on its location: the peripheral bone bridges can be approached directly while the central bone bridges can be approached either through a metaphyseal window, or through a metaphyseal osteotomy and curettage of the cancellous bone up to the bone bridge. A high-speed burr is usually used for complete removal of the bone bridge (10).

The spatiotemporal growth factor control model of bone elongation has been held to be majorly responsible for the growth plate stimulus in the past two decades (11). However, advances in articular cartilage biology have turned the attention to the potential regulation of growth by mechanical forces. Indeed, several recent papers have presented evidence supporting a primary role of mechanical forces in promoting normal growth plate cartilage function. It would seem in fact that additional compressive stress avoided bone growth, whereas reducing compressive stress enhances bone growth, with differences between static and dynamic mechanical forces (11). The initial goal of our treatment was to stabilize the deformity during the further phases of growth and then to indicate a possible corrective osteotomy. The interest of our case lies in the fact that the removal of the bone bridge was more than 30% of the entire growth plate area. In the initial stages, the presence of bone wax in the area of de-epiphysiodesis allowed the stabilization of the deformity on the 20° of preoperative valgus. The interpretation of the growth cartilage activity occurred, during the adolescent spurt of growth, in an asymmetrical way such as to realign the femoral load axis, it can be based on the different mechanical stimulus on the two knee areas (medial and lateral) due to the preexisting deformity. The chondrocytes of the lateral zone, receiving a different mechanical stimulus, responded with a hyperactivity up to the recovery of the joint plane and, therefore, of a similar mechanical stimulus in the two areas. Ultimately, the outcome of the treatment was a 20 mm discrepancy, which could lead in the long term, if not compensated, to recurrent low back pain due to overload. Furthermore, if we had not obtained the 20° valgus correction, the patient would have risked an overload of the lateral compartment with an increased risk of long-term osteoarthritis.

There is no unanimous evidence in the literature in terms of the management of growth disorders resulting from this type of injury. With fractures at high risk of complications, such as type IV SH or distal femur fractures, an acute Langenskiöld procedure could prevent bone bar formation (12). When the formation of bone bars occurs in patients with potential growth disorders, the current gold standard therapy is the resection of the bone bar with subsequent interposition of materials such as free fat graft, PMMA, silastic, cartilage, bone wax, and dura (2). Bone wax resulted in effectively filling the hole of de-epiphysiodesis in distal femoral growth plate and allowed us to obtain the response of the growth plate and to improve the recovery time in a young child. Future treatment alternatives include treatments ranging from physeal transplantation to regenerative and tissue-engineering approaches (13).

## Conclusion

To the best of our knowledge, this is the first case described in the literature of long-term follow-up of complex injuries of physis of the knee in 5-year-old patient involved in crushing polytrauma. Early diagnosis and physeal-sparing techniques are important but not sufficient to prevent future physeal growth arrest and resultant growth disturbances.
